# Effect and underlying mechanisms of airborne particulate matter 2.5 (PM2.5) on cultured human corneal epithelial cells

**DOI:** 10.1038/s41598-020-76651-9

**Published:** 2020-11-11

**Authors:** Kenji Kashiwagi, Yoko Iizuka

**Affiliations:** Department of Ophthalmology, University of Yamanashi, Chuo, Yamanashi Japan

**Keywords:** Environmental chemistry, Corneal diseases

## Abstract

Health problems caused by airborne particulate matter with a diameter less than 2.5 (PM2.5), especially in the respiratory system, have become a worldwide problem, but the influence and mechanisms of PM2.5 on the ocular surface have not been sufficiently elucidated. We investigated in vitro the onset and pathogenesis of corneal damage induced by PM2.5. Two types of PM2.5 samples originating from Beijing (designated #28) and the Gobi Desert (designated #30) were added to the culture medium of immortalized cultured human corneal epithelial cells (HCECs) to examine the effects on survival rates, autophagy, and proinflammatory cytokine production. Both types of PM2.5 significantly reduced the HCEC survival rate in a concentration-dependent manner by triggering autophagy. In particular, compared with #30, #28 induced much more severe damage in HCECs. Physical contact between PM2.5 and HCECs was not a primary contributor to PM2.5-induced HCEC damage. Among the 38 proinflammatory cytokines examined in this study, significant increases in the granulocyte macrophage colony-stimulating factor (GM-CSF) and interleukin-6 levels and a significant reduction in the interleukin-8 level were detected in culture medium of PM2.5-exposed HCECs. Simultaneous addition of a GM-CSF inhibitor, suramin, alleviated the HCEC impairment induced by PM2.5. In conclusion, PM2.5 induces HCEC death by triggering autophagy. Some cytokines that are released from HCECs, including GM-CSF, may be involved in HCEC damage caused by PM2.5 exposure.

## Introduction

Air pollution-related adverse health effects have become a major problem in recent years. Small particulate matter with a diameter less than 2.5 µm (PM2.5) is particularly deadly, with a 36% increase in lung cancer per 10 μg/m^3^ because it can penetrate deep into the lungs^1^. PM2.5 exposure contributed to 4.1 million deaths worldwide from heart disease, stroke, lung cancer, chronic lung disease, and respiratory infections in 2016^2^.

Airborne particulate matter comes in a variety of sizes, but depending on its size, it behaves differently in the body and has different health effects. The magnitude of health disturbance depends on the particulate matter concentrations, and particle size is correlated with the severity of health damage. Many studies have suggested that particulate matter, especially PM2.5, increases morbidity or mortality related to cardiovascular and pulmonary diseases^3,4^. Since the ocular surface is constantly exposed to the external environment, it may represent a good model of PM2.5-induced adverse health effects. Indeed, recent studies have shown that PM2.5 has adverse effects on the human ocular surface, including the cornea and conjunctiva^5–7^. High concentrations of air pollutants frequently result in ocular symptoms such as burning, itchiness, and redness^8^. Moreover, PM2.5 may exacerbate the development of allergic conjunctivitis and other ocular surface diseases^6,7,9,10^.

Recent studies have revealed that autophagy, proinflammatory cytokines, oxidative stress, and mitochondrial damage are complications involved in PM2.5-induced adverse health effects^11–17^. Autophagy inhibitors have been reported to eliminate or alleviate PM2.5-induced symptoms^11^. Whether PM2.5 exerts similar effects on the ocular surface and the identity of the molecular mechanisms involved in those effects are unclear; however, previous studies reported that autophagy and proinflammatory cytokines play important roles in PM2.5-induced adverse effects on corneal epithelium cells^13,18^.

There are two main purposes in this study. First, we elucidated the effect and mechanism of action of PM2.5 on cultured human corneal epithelial cells (HCECs). Second, PM2.5 contents may differ among PM2.5 samples. Indeed, two PM2.5 samples derived from Beijing and the Gobi Desert had a different content profile, as Supplementary Table 1 shows. PM2.5 impairment may vary among previous reports, and differences in PM2.5 contents may result in different health impairments. We employed two types of PM2.5 samples that have been analyzed for their contents to elucidate the mechanism of PM2.5-related damage in HCECs.

## Results

### Effects of PM2.5 on the survival rate and morphological features of HCECs

**Figure 1:**
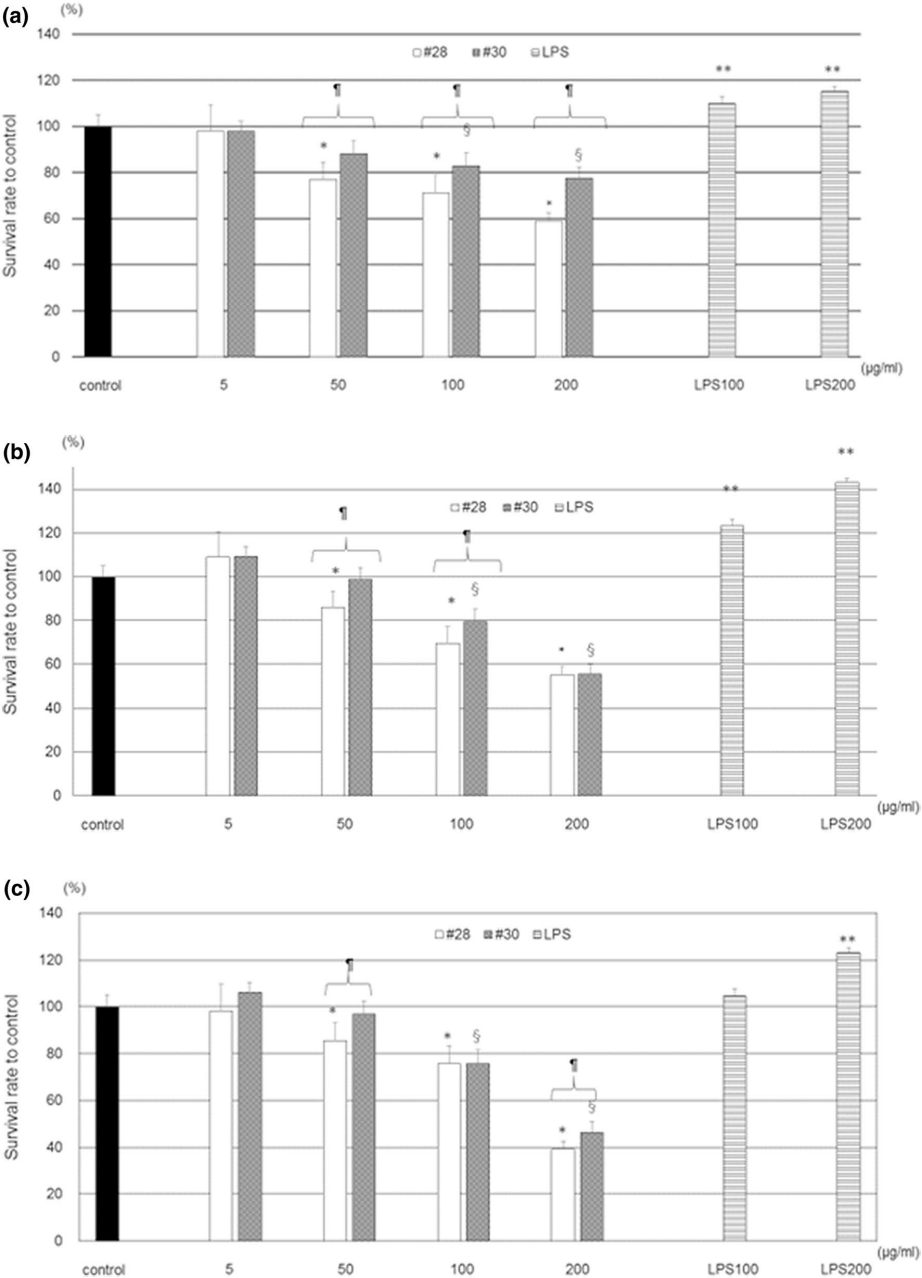
Effect of PM2.5 on HCEC survival. Exposure to PM2.5 resulted in a concentration-dependent reduction in HCEC survival after 4 (**a**), 24 (**b**), and 48 (**c**) hours of exposure. Both #28 and #30 caused significant dose-dependent reductions in HCEC survival rates. *A significant decrease relative to the control and 5 μg/ml #28, ^§^A significant decrease compared to the control and 5 μg/ml #30, ^¶^A significant difference between #28 and #30, **A significant difference compared with the control and all concentrations of #28 and #30, n = 8–10, *LPS *lipopolysaccharide.

Phase-contrast microscopy revealed shrinkage of cells after 4 h of exposure to 2 types of PM2.5, and a significant reduction in the survival rate was observed after 24 h of exposure. In contrast, LPS exposure resulted in a significant increase in the survival rate, and HCECs tended to proliferate after LPS exposure (Fig. 1 and Supplementary Fig. 1).

The HCEC survival rate was significantly affected by exposure to both types of PM2.5 in a concentration-dependent manner. Comparing the two PM2.5 samples, the survival rate of HCECs exposed to #28 was significantly lower than that of HCECs exposed to #30, especially after 4 h of exposure at all concentrations and after 48 h of exposure at concentrations of 50 and 200 μg/ml.

### Effect of PM2.5 extract on HCECs evaluated using PM2.5 supernatant and a noncontact coculture system containing HCECs and PM2.5

**Figure 2:**
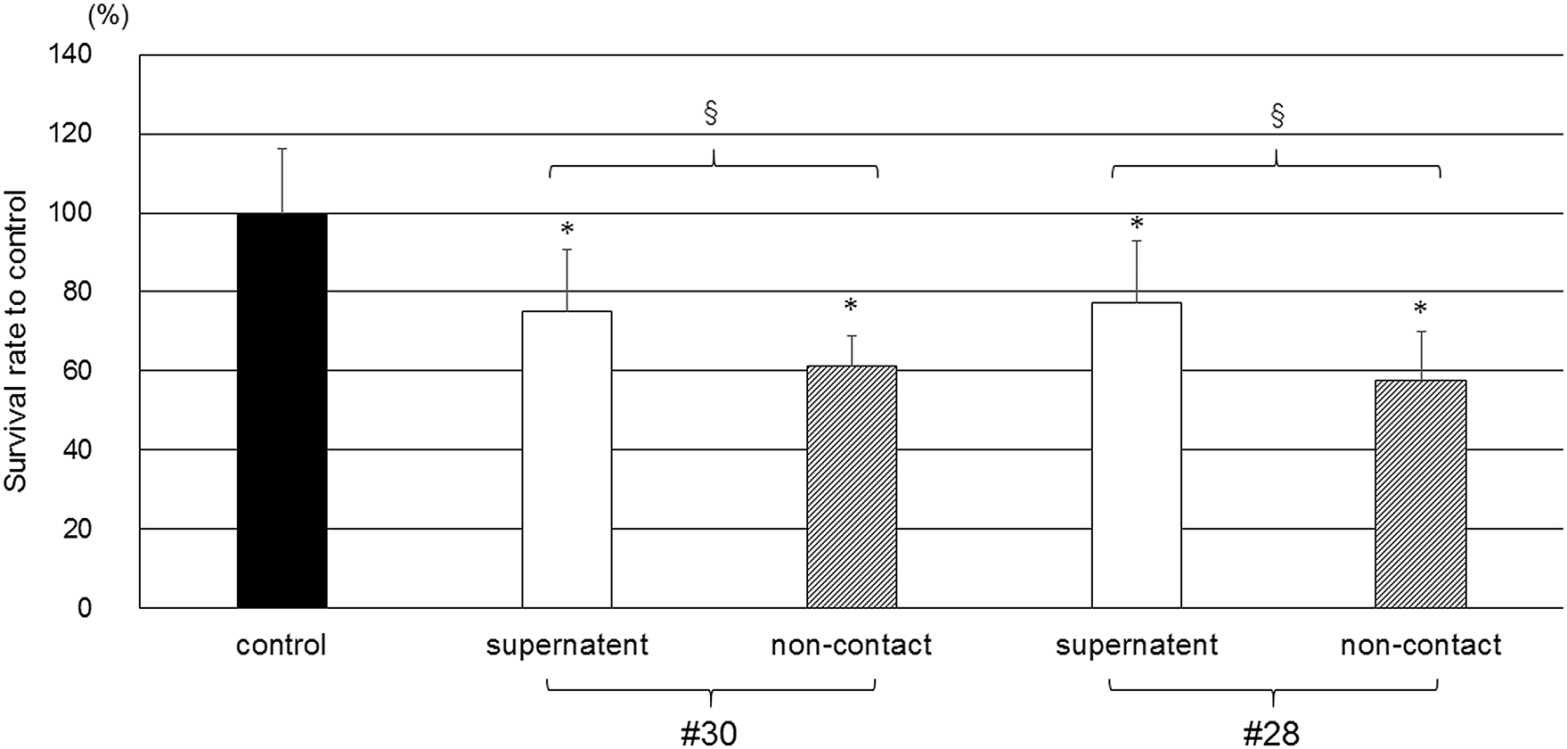
Effects of PM2.5 extract on HCECs determined using the supernatant from PM2.5 and a noncontact coculture system containing HCECs and PM2.5. The concentrations of PM2.5 were 100 μg/ml in #28 and #30 and 0 μg/ml in the control. *A significant decrease relative to the control, ^§^A significant difference between #28 and #30, n = 10.

Compared with the supernatant from the controls, supernatants from both #28 and #30 significantly decreased the survival rate of HCECs. There was no significant difference in the survival rate of HCECs between the #28 and #30 exposure groups. The survival rate of HCECs cultured in a noncontact coculture system was significantly lower than that of HCECs cultured with the supernatant in both the #28 and #30 exposure groups (Fig. 2). The coculture system did not influence the survival rate of HCECs (Supplementary Fig. 2).

### PM2.5-induces autophagy

**Figure 3:**
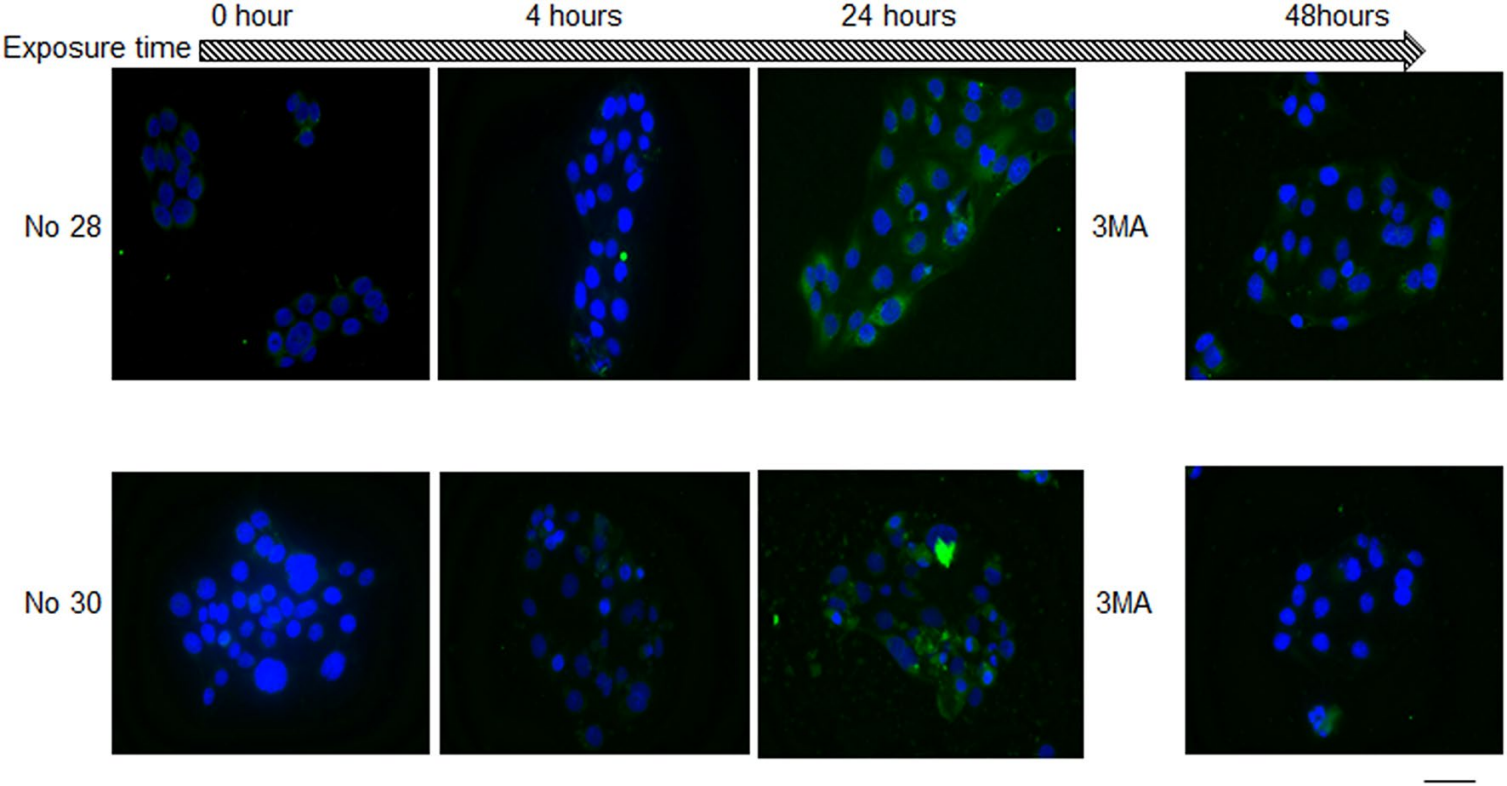
Observation of PM2.5-induced autophagy by fluorescence microscopy. The levels of autophagy induced by PM2.5 were augmented by both types of PM2.5 in proportion to the loading time. The autophagy levels gradually increased with exposure time compared with that at baseline. Autophagy induction was blocked by concomitant 3-MA administration. Green cell body staining indicates autophagy. Cell nuclei were counterstained blue with 4′,6-diamidino-2-phenylindole. *3-MA* 3-methyladenine. Scale bar = 100 µm.

Fluorescence microscopy revealed that compared with the control, both #28 and #30 PM2.5 increased the level of autophagy. Fluorescein intensity in HCECs increased with increasing exposure time to #28 and #30 (Fig. 3).

**Figure 4:**
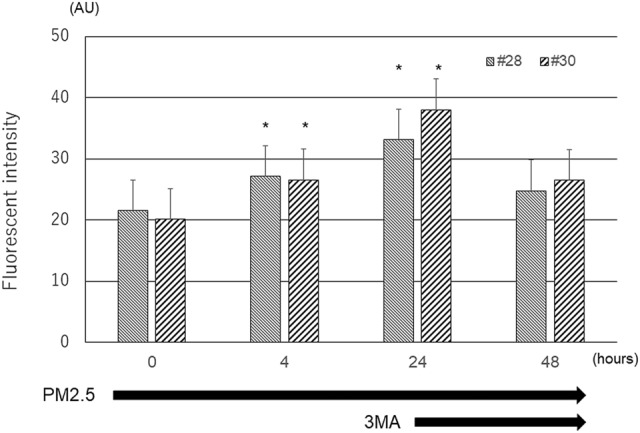
Quantitative analysis of PM2.5-induced autophagy in HCECs. PM2.5 significantly increased the level of autophagy activity in HCECs in proportion to the loading time, and additionally applied 3-MA inhibited the elevation of autophagy activity. There was no significant difference in the change in autophagy after exposure to the two types of PM2.5. *p < 0.01 vs. control, bar = SD, n = 6.

The results of the semiquantitative expression analysis confirmed that the degree of autophagy was significantly elevated in the #28 and #30 exposure groups compared to that in the control group. However, there was no significant difference in the levels of autophagy between the #28 and #30 exposure groups (Fig. 4).

When 10 mM 3-MA was administered at the same time as #28 or #30, the fluorescence intensity due to autophagy was reduced (Figs. 3, 4).

### Measurement of cytokine levels

Screening identified five types of cytokines with concentrations above the limit of quantification, namely, tumor necrosis factor-α (TNF-α), granulocyte macrophage colony-stimulating factor (GM-CSF), interleukin-2 (IL-2), interleukin-6 (IL-6) and interleukin-8 (IL-8). The changes in the concentrations of these five cytokines were inconsistent, and the concentrations of the 38 cytokines are shown in Supplementary Table 2.

### Effects of PM2.5 exposure on the expression of five selected cytokines

**Figure 5:**
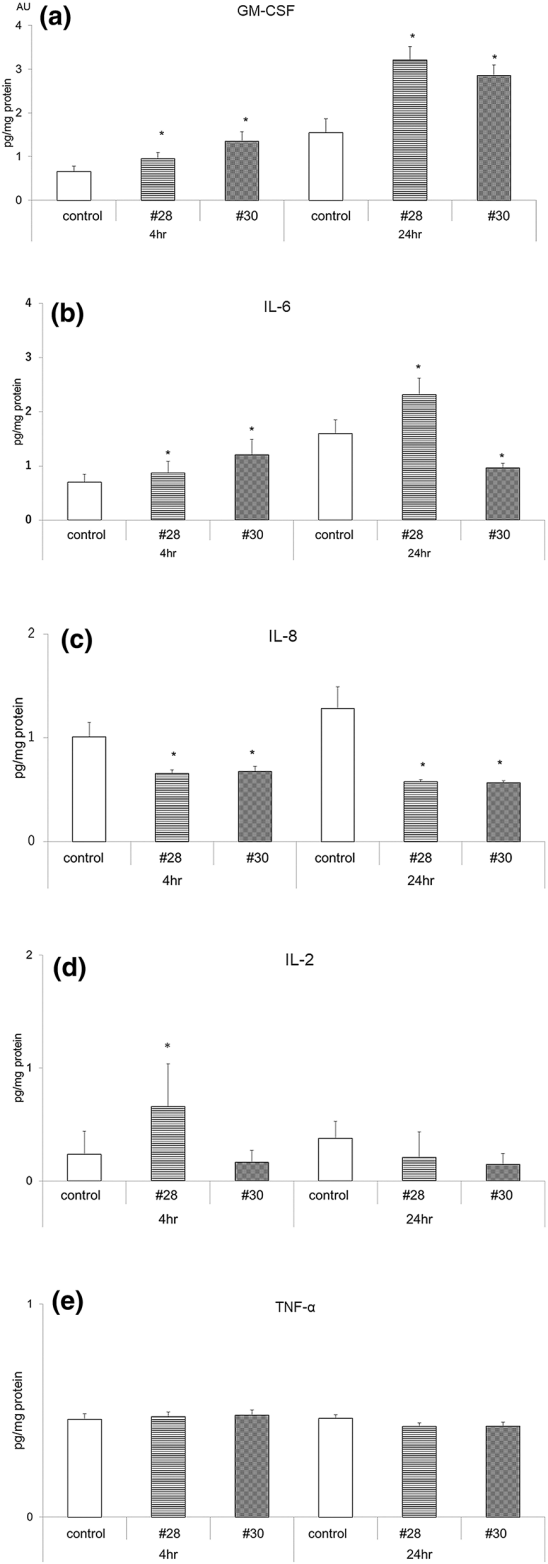
Changes in the levels of cytokines released from cultured corneal epithelial cells. Changes in the release of GM-CSF (**a**), IL-6 (**b**), IL-8 (**c**), IL-2 (**d**), and TNFα (**e**) into the culture medium. n = 6, *p < 0.01 vs. control. *GM-CSF* granulocyte macrophage colony-stimulating factor, *IL* interleukin, *TNF* tumor necrosis factor.

Changes in the concentrations of the five selected cytokines following PM2.5 exposure varied depending on the individual cytokine. Furthermore, the changes in cytokine concentrations in response to exposure to #28 and #30 were not the same. The concentration of GM-CSF was significantly increased at 4 h and 24 h after exposure compared with that of the control; after 24 h of exposure, the GM-CSF concentration was approximately twice that of the control. There was no significant difference in the concentrations of GM-CSF based on the PM2.5 type (#28 and #30, Fig. 5a). The IL-6 concentration was significantly elevated at 4 h of exposure to #28, but it was significantly reduced after 24 h of exposure to #30 (Fig. 5b). The IL-8 concentration was significantly lower after exposure to PM2.5 compared with that of the control at all time points (Fig. 5c). The IL-2 concentration was significantly increased after 4 h of exposure to #28, but no significant differences were observed at the other time points (Fig. 5d). With regard to the TNF-α concentration, no significant differences between the control and exposure groups were observed at any time point (Fig. 5e).

### Role of GM-CSF and IL-6 in PM2.5-induced HCEC death (Fig. 6)

**Figure 6:**
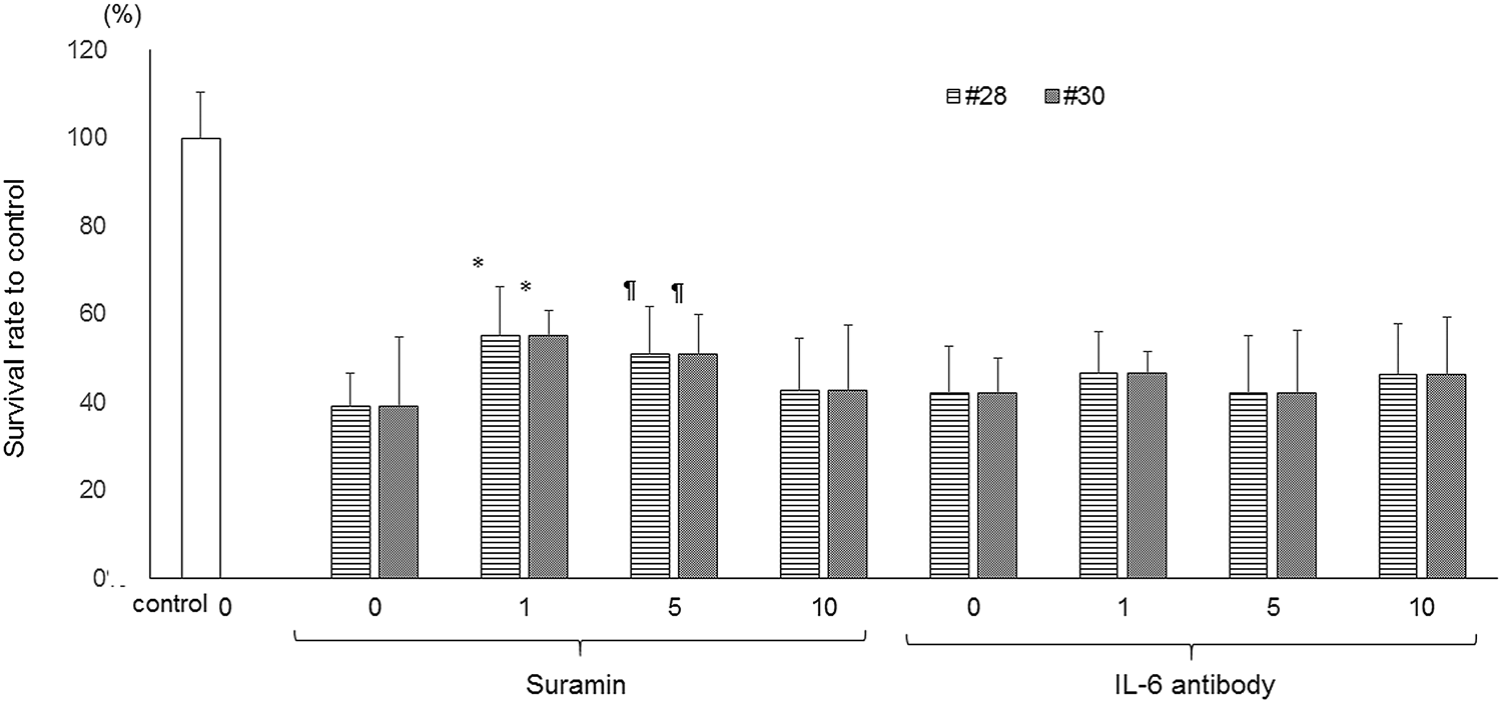
Effects of suramin and an IL-6 antibody on PM2.5-induced HCEC damage. n = 6–10, *p < 0.01, ^¶^ < 0.05 vs. control, *IL *interleukin.

Compared with exposure to PM2.5 alone, simultaneous exposure to 1 or 5 μM suramin and PM2.5 partially but significantly enhanced HCEC survival. With an increase in the concentration of suramin, the level of inhibition of PM2.5-induced HCEC death was reduced. There was no significant difference in the suramin-related protective effect on PM2.5-induced HCEC death between #28 and #30. In contrast, simultaneous administration of an anti-IL-6 antibody with PM2.5 did not influence HCEC death. Compared with administration of GM-CSF alone, addition of suramin and anti-IL-6 antibodies concomitant with exposure to PM2.5 resulted in no additional effect on the survival rate of HCECs.

## Discussion

The current study revealed that two PM2.5 samples induced HCEC death and morphological changes in a concentration-dependent manner. However, the effects of these samples on HCECs were not the same. Although direct physical damage to HCECs induced by PM2.5 cannot be avoided, the current study indicated that the components of PM2.5 are at least partially responsible for inducing HCEC death. Autophagy is an important mechanism underlying PM2.5-induced HCEC death, and some cytokines, especially GM-CSF, are involved in HCEC death.

Previous reports have investigated the influence of PM2.5 on corneal cells. Some previous reports using different cell resources showed that PM2.5 results in corneal epithelium death in a concentration-dependent manner, which is similar to the results of the current report^18,19^. The mechanisms underlying PM2.5-induced cell damage have been previously investigated. Yoon et al. reported that the inflammatory response, mitochondrial activity, and oxidative stress participate in cell death^18^, and Gao et al. found that reactive oxygen species and DNA damage result in corneal epithelial cell senescence following exposure to PM2.5 collected in Guangzhou^11,19^. Moreover, expression changes in cancer genes (oncogenes) are caused by PM2.5 exposure^12,20^. Cui et al. reported that disturbances to the FAK/RhoA signaling pathway and cytoskeletal organization resulting from PM2.5 exposure damaged mouse corneal epithelial cells, using a scratch model and an in vitro experimental system^21^.

As some studies have shown the involvement of proinflammatory cytokines in the cytotoxicity induced by PM2.5, we investigated changes in the concentration of a variety of cytokines in the current study. Notably, some proinflammatory cytokines may be partially involved in HCEC death through their involvement in autophagy, and some previous studies investigated the relationship between proinflammatory cytokines and cell damage induced by PM2.5 exposure using different types of cells. For example, Li et al. exposed rat lungs for 9 days to concentrations of PM2.5 ranging from 0.375 to 24.0 mg/kg by intratracheal instillation^22^. As a result, the mRNA and protein concentrations of TNF-α, IL-6, IL-1β, and ICAM-1 were elevated after exposure to concentrations of 6 mg/kg or greater^12,22^. Yoon et al. reported an increase in the concentration of IL-8 in cultured HCECs induced by PM2.5 exposure^18^. The authors compared PM2.5-induced changes in IL-8 among different culture conditions. Exposure to 100 μg/ml PM2.5 resulted in the largest increase in the IL-8 concentration in HCECs, while the supernatant from 500 μg/ml PM2.5 or a pellet containing 10 μg/ml PM2.5 resulted in the largest increase in the concentration of IL-6 released from HCECs. The increase in the IL-6 concentration in the current study was consistent with the findings in previous reports. However, the lack of changes in the TNF-α concentration, the reduction in the IL-8 concentration, and the lack of detectable IL-1β levels were inconsistent with the results of previous studies. Some reasons for these differences could include the cultured cell type, PM2.5 samples employed, PM2.5 loading amounts, or experimental conditions, such as in vitro versus in vivo.

The relationship between autophagy and cytokines is controversial. Autophagy system malfunction is thought to cause the overexpression of inflammatory cytokines, while autophagy promotes the secretion of inflammatory cytokines, such as IL-1β and IL-18^23^. The current study provided new insight into the roles played by cytokines in PM2.5-induced HCEC death. First, the current study showed a significant increase in GM-CSF expression in HCECs, and GM-CSF inhibition at least partially alleviated PM2.5-induced HCEC death. In contrast, IL-6 inhibition did not influence PM2.5-induced HCEC death, although the IL-6 concentration was also significantly elevated following PM2.5 exposure. Some previous studies have reported this phenomenon in pulmonary epithelial cells^14,24–26^. Rho et al. reported that GM-CSF promotes corneal epithelium migration and contributes to wound healing but that it does not affect proliferation^27,28^. Epithelial cell-derived GM-CSF reportedly contributes to the intrinsic defense mechanisms against lung injury^29^. Blanchet et al. reported that PM2.5 induces GM-CSF through the upregulation of amphiregulin expression and secretion^14^. Amphiregulin is an EGFR ligand that may be involved in an important mechanism that sustains the proinflammatory response. However, the findings of these previous reports are inconsistent with the current results. Although we employed suramin as a GS-CSF inhibitor, suramin has a variety of other effects in addition to GS-CSF inhibition, which may have influenced the impairment of HCECs by PM2.5. Moreover, PM2.5 influenced the secretion of several types of cytokines in addition to that of GS-CSF. Other mechanisms may exist, given the limited contribution of the mechanism revealed in this study. It is known that there are complicated feedback loops between the autophagy pathway and inflammation^30^.

We compared two samples of PM2.5 from Beijing and the Gobi Desert. Although both samples exerted similar effects on HCECs in terms of the changes in cytokine expression and the degree of autophagy, compared with #30, #28 showed a significantly greater impact on the survival rate of HCECs in the current study. One of the reasons for this difference is that the autophagy measurements may not have been able to detect the difference between the two PM2.5 effects due to the relatively wide range of variation. The two types of PM2.5 used in this study contain a wide variety of components, as indicated in Supplementary Table 1, which may be complexly related to HCEC death through actions other than autophagy. It is difficult to identify which component accounts for this different effect, though compared with #30, #28 contains more types of highly toxic components, such as Ni, Cd, and Pb. Further analysis of the components responsible for the difference in cytotoxicity between the two samples and a survey of the components would be useful.

The current study employed an in vitro system using isolated immortalized HCECs to investigate the effects of PM2.5. Under in vivo conditions, HCECs coexist with other cell types and the extracellular matrix. Exposure to ambient air pollution levels could affect the conjunctival goblet cell density^6^. In this study, purely isolated HCECs were cultured in serum-free medium at the time of their exposure to PM2.5, which may have affected the cellular responses. Moreover, under in vivo conditions, PM2.5 may react directly with human tissues, including the ocular surface. We prepared dissolved PM2.5 in this study, which may lead to different results than those obtained under in vivo conditions. Therefore, we cannot directly apply these results to individuals.

The loading conditions used in this study may not be the same as those under conditions of ambient air pollution. However, the annual average and maximum concentrations of PM2.5 in the atmospheric air in Beijing in 2016 were 71 μg/m^3^ and 150 μg/m^3^ (usually in the winter season), respectively. These concentrations are quite high compared to the annual average environmental standard of 10 μg/m^3^ PM2.5 reported by the World Health Organization (WHO)^31^. Since the concentration of PM2.5 in tears is not available, it is currently impossible to conclude whether the concentrations used in this study are reasonable. However, it is better to avoid exposure of the ocular surface to high concentrations of PM2.5, especially for individuals with reduced abilities to produce tears and maintain a tear film on the ocular surface.

In the current study, two different types of PM2.5 exerted similar but not identical cytotoxic effects on HCECs, and the involvement of autophagy and proinflammatory cytokines in the cytotoxic effect was clear. Since these effects depend on the type of PM2.5, further investigations using different samples collected from a variety of regions are necessary.

## Materials and methods

### PM2.5 samples

PM2.5 is defined by only the size of the particles and not by its composition. The impairment caused by PM2.5 has not been constant among studies, and the different PM2.5 components used in each study may affect the results. For this reason, we used two environmental standard substances that were refined and managed by the National Institute for Environmental Studies (NIES). One substance was atmospheric dust derived from Beijing (NIES CRM No. 28, referred to as #28), and the other substance was atmospheric dust derived from the Gobi Desert (NIES CRM No. 30, referred to as #30). We present the details of the components of these two PM2.5 samples in Supplementary Table 1.

### Human corneal epithelial cells

HCECs (#RCB1384) were provided by RIKEN BRC CELL BANK (Tsukuba, Ibaraki, Japan). The cells were cultured in DMEM/F12 (Gibco, CA) with 15% fetal bovine serum (FBS) (Gibco) and passaged with 0.25% trypsin and 0.02% ethylenediaminetetraacetic acid (EDTA) (Gibco) every three days. The HCECs were then seeded onto culture plates overnight to allow attachment before PM2.5 treatment. Subsequently, all culture medium was replaced with fresh medium containing a PM2.5 suspension supplemented with 100 U/ml penicillin and 0.1 mg/ml streptomycin (Gibco).

### Effect of PM2.5 on HCEC survival rates

Initially, we determined the proper PM2.5 concentrations according to methods in previous reports^18,19,21^. The annual average PM2.5 concentration in the atmosphere in the urban Beijing area during previous years was approximately 100 μg/m^3^, and previous in vitro reports have employed a series of concentrations of PM2.5 from 10 to 500 μg/ml^13,18^. Therefore, we used 5, 50, 100, and 200 μg/ml PM2.5 in this study. According to the method described by Fu et al., HCECs were seeded onto 96-well culture plates at a density of 5 × 10^3^ cells/well. After an initial 24-h culture period, the culture medium was replaced with fresh medium containing the final concentrations of 5, 50, 100, and 200 μg/ml PM2.5. Two controls were prepared, namely, medium without PM2.5 and medium with final concentrations of 100 or 200 μg/ml lipopolysaccharide (LPS). The cell survival rate and morphological changes were evaluated 4 h, 24 h, and 48 h after exposure. The survival rate was determined using a Cell Counting Kit-8 assay (Dojindo Molecular Technologies, Kumamoto, Japan) according to instructions in the user’s manual. This assay measured the amount of formazan dye generated by cellular dehydrogenase activity, which is directly proportional to the number of living cells. In brief, we prepared a dilution series of HCECs cultured for up to 48 h as a preliminary experiment to create a standard line with an absorbance ranging from 0.5 to 2 AU. Then, the cell concentration suitable for measurement was determined. Next, the HCECs used for measurements were adjusted such that the final cell number was 5 × 10^3^ cells/culture well; the cells were seeded on a 96-well plate and cultured as scheduled. Ten microliters of Cell Counting Kit-8 solution was added to each well, and the color reaction was allowed to proceed for 2 h in a CO_2_ incubator. Absorbance at 415 nm was measured with a microplate reader. The survival rate was determined based on the absorbance of the vehicle control without the addition of PM2.5 compared with the absorbance of the cells with the addition of PM2.5. Changes in HCEC morphology after PM2.5 exposure were observed with a phase-contrast microscope (ECLIPSE TE300, NIKON, Tokyo, Japan).

### Effect of PM2.5 extract on HCECs determined using supernatants of PM2.5 and a noncontact coculture system containing HCECs and PM2.5

The following two conditions were examined to determine whether the effect of PM2.5 on HCECs is due to the physical action of PM2.5 or its constituents. We cultured HCECs under the following three conditions. (1) First, 100 μg/ml PM2.5 was incubated for 24 h at 37 °C in medium without HCECs to generate a culture medium supernatant containing PM2.5 components. Then, the collected medium was replaced with culture medium in which HCECs had been cultured for 24 h, and another 24-h incubation was performed. Since it is possible that the release of contained material from PM2.5 to the culture medium may be influenced by the presence of HCECs, the following experimental conditions were created. (2) HCECs were similarly seeded in a 24-well culture dish and cultured for 24 h. PM2.5 was placed on an insert (Millicell-CM; membrane area, 0.6 cm^2^; pore size, 0.4 μm; Millipore, Bedford, MA) and incubated for another 24 h. (3) To investigate the influence of the insert on HCEC survival, HCECs were seeded in a 24-well culture dish under conditions similar to those in (1) and cultured for 24 h. Then, the insert was placed, and the HCECs were cultured for an additional 24 h. The survival rate of the HCECs was evaluated using a Cell Counting Kit-8 assay.

### Role of autophagy in PM2.5-induced HCEC damage

Some previous studies report that autophagy is involved in cell death induced by PM2.5^4–6,12,32^, and Fu et al. reported autophagy induced by PM2.5 in HCECs^1,13^. Since the HCECs used by Fu et al. were different from those used in this study, we confirmed the role of autophagy in HCEC damage induced by PM2.5 in this experiment. Autophagy was examined using a Cell Meter autophagy fluorescence imaging kit (# 23002, AAT Bioquest, CA). This kit employs a specific autophagosome marker to analyze autophagy activity. The experiment was conducted according to the instructions in the user’s manual. HCECs were cultured on 8-well glass plates overnight, and then the experiment was initiated. In total, 1.134 × 10^4^ cells/500-μl well were incubated with #28 or #30 for 24 h at 37 °C.

Based on the survival rates of the HCECs following PM2.5 exposure, the optimal concentrations of #28 and #30 were determined to both be 100 μg/ml. The status of autophagy was evaluated at 4 and 24 h of exposure to PM2.5. To confirm changes in autophagy, we simultaneously added the autophagy inhibitor 3-methyladenine (3-MA) (M9281, Merck KGaA, Darmstadt, Germany), a class III PI3-kinase inhibitor, and 100 μg/ml #30 or #28. The 3-MA concentration was set to 10 mM according to a previous report^4,5,28,33^. Cells undergoing autophagy were identified as cells with a bright fluorescent signal in the cytoplasm. Microscopic observation and quantitative analysis of autophagy were performed with a fluorescence microscope (KEYENCE, BZX 700, Osaka, Japan).

### Measurement of cytokines

Although some previous studies indicated that several cytokines are involved in cell damage induced by PM2.5^11,16^, their role in HCEC damage has not been fully identified, and the types of cytokines investigated were limited. For these reasons, we evaluated a wide variety of cytokines involved in HCEC damage in this study. We performed two consecutive experiments to screen and quantify the cytokines expressed in the medium. To screen the cytokines, the concentrations of 38 cytokines were measured using a multiplex map human cytokine/chemokine magnetic bead panel (cat # HCYTMAG-60K-PX38, Merck KGaA). In total, 1.134 × 10^4^ HCECs/500-μl well were incubated for 24 h in DMEM/F12 with 5% FBS. Then, the medium was replaced by serum-free medium with 100 μg/ml #28, 100 μg/ml #30, or the vehicle control for an additional 24-h incubation at 37 °C. The medium was then collected and centrifuged at 13,000 × *g* at 4 °C for 5 min, and the supernatant was recovered. Each sample solution was sent to Genetic Lab Inc. (Sapporo, Japan) for analysis. The cytokine concentrations in each sample were determined in duplicate.

Some cytokines with reliable concentrations in the screening experiment were subjected to quantitative analysis. After an initial 24-h incubation, the HCEC medium was replaced with fresh medium containing 100 μg/ml #28, 100 μg/ml #30, or the vehicle control for another 4 h or 24 h of incubation at 37 °C. The culture medium in the wells was collected and centrifuged at 13,000 × *g* at 4 °C for 5 min, and the supernatants were sent to Genetic Lab Inc. for measurement of the concentrations of the selected cytokines using the same multiplex map human cytokine/chemokine magnetic bead panel as before.

### Role of selected cytokines in HCEC damage

In the cytokine quantification experiments, there were significant differences in the concentrations of GM-CSF and IL-6 between PM2.5-exposed HCECs and the control cells. Therefore, these two cytokines were selected for the investigation of the role of cytokines in PM2.5-induced HCEC damage.

Changes in cell damage were examined after adding GM-CSF and IL-6 inhibitors, which were selected on the basis of their marked differences in concentrations between the control and PM2.5-treated groups. After the HCECs were cultured in 5% FBS in DMEM/F12 at 37 °C for 24 h, the culture medium was replaced by fresh medium containing 100 μg/ml #28 or #30, 100 μg/ml #28 or #30 with 0, 1, 5, or 10 μM suramin, or the vehicle control for an additional 24-h incubation. The survival rate of HCECs after culturing was determined using a Cell Counting Kit-8 assay. To investigate the role of IL-6 in PM2.5-induced HCEC damage, 0, 1, 5 or 10 μg/ml anti-IL-6 antibody (Sigma cat # 17901, Merck KGaA) was added to the medium instead of suramin. The HCEC survival rate after 24 h of culturing was also determined using a Cell Counting Kit-8 assay.

### Statistics

For the comparisons of survival rates, autophagy activity, and cytokine concentrations among the experimental conditions, one-way ANOVA and the Tukey–Kramer honesty significant difference (HSD) test were employed. The significance level was set at p < 0.05. The results are expressed as the means ± standard deviations.

## Supplementary information

Supplementary Information 1.

Supplementary Information 2.

## Data Availability

The datasets generated during and/or analyzed during the current study are available from the corresponding author on reasonable request.
