# Supplementary material for: Effect and underlying mechanisms of airborne particulate matter 2.5 (PM2.5) on cultured human corneal epithelial cells
Source: Sci Rep. 2020 Nov 11;10:19516. doi: 10.1038/s41598-020-76651-9 (PMC7659009; doi:10.1038/s41598-020-76651-9)
Supplement: Supplementary file 1 — Supplementary Information 1. [file 41598_2020_76651_MOESM1_ESM.pdf]

Supplemental Figure 1
Title: Effect and underlying mechanisms of airborne particulate matter 2.5 (PM2.5) on cultured human corneal epithelial cells
Authors: Kenji Kashiwagi and Yoko Iizuka

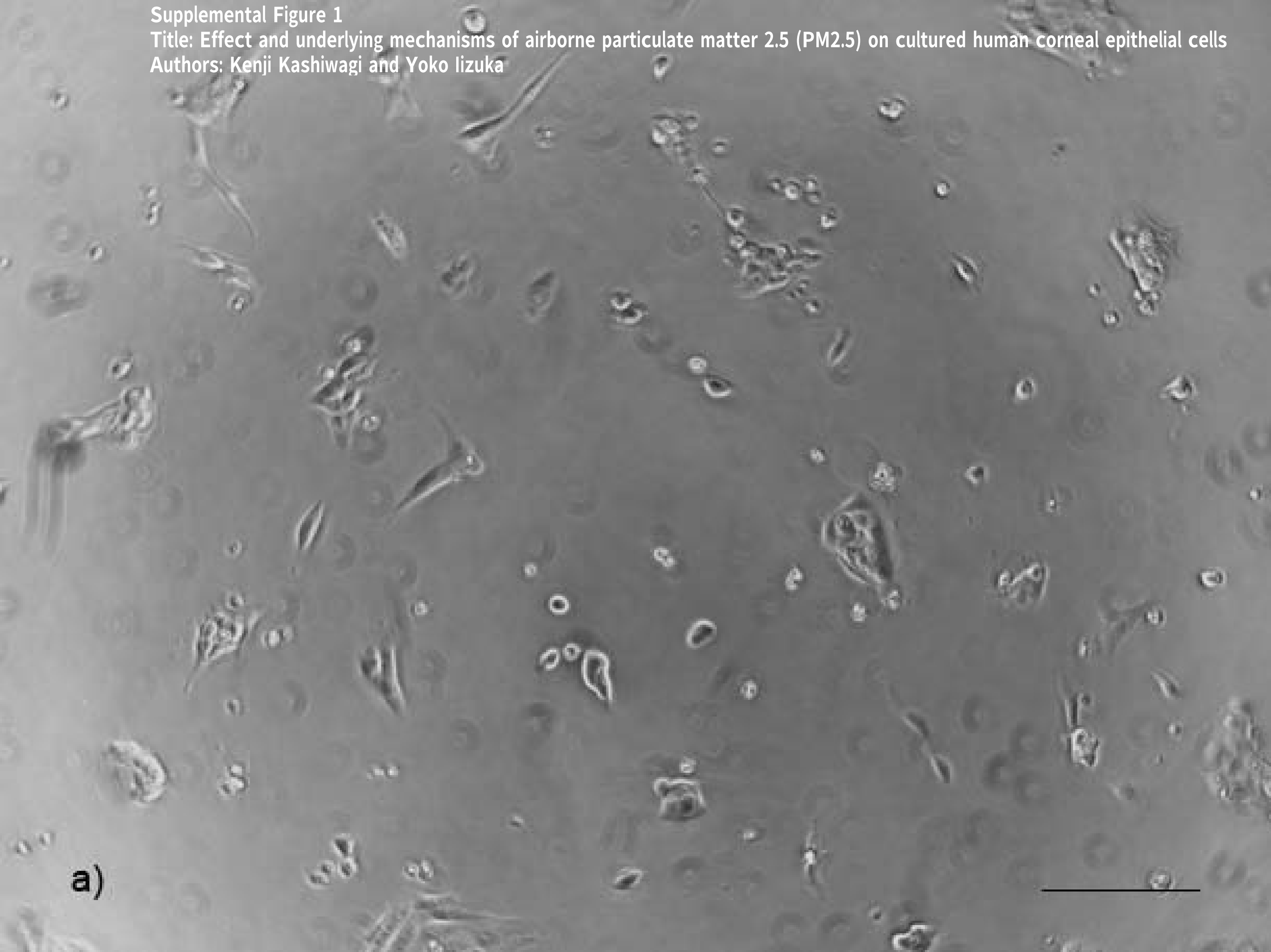

Supplemental Figure 1
Title: Effect and underlying mechanisms of airborne particulate matter 2.5 (PM2.5) on cultured human corneal epithelial cells
Authors: Kenji Kashiwagi and Yoko Iizuka

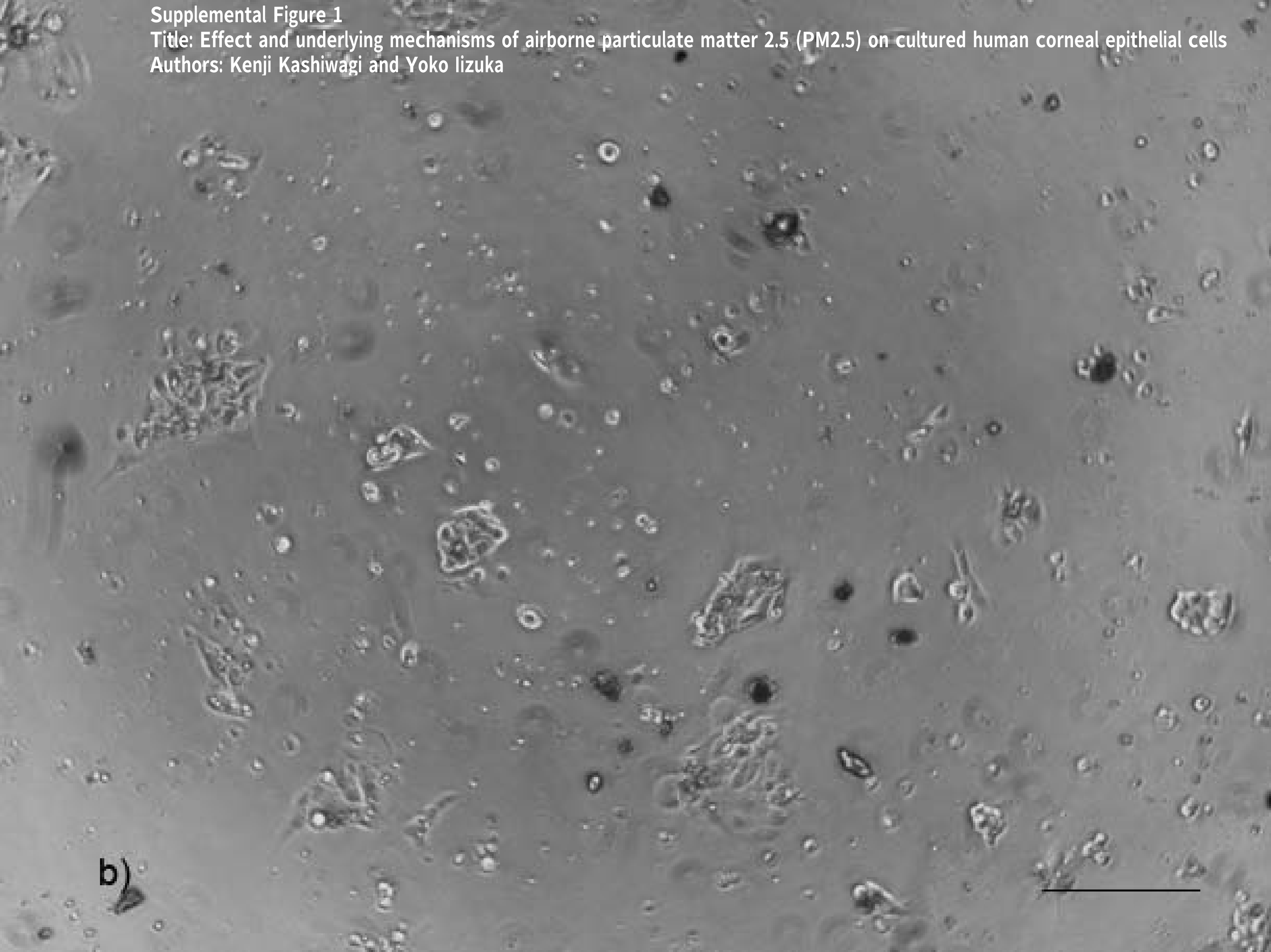

Supplemental Figure 1
Title: Effect and underlying mechanisms of airborne particulate matter 2.5 (PM2.5) on cultured human corneal epithelial cells
Authors: Kenji Kashiwagi and Yoko Iizuka

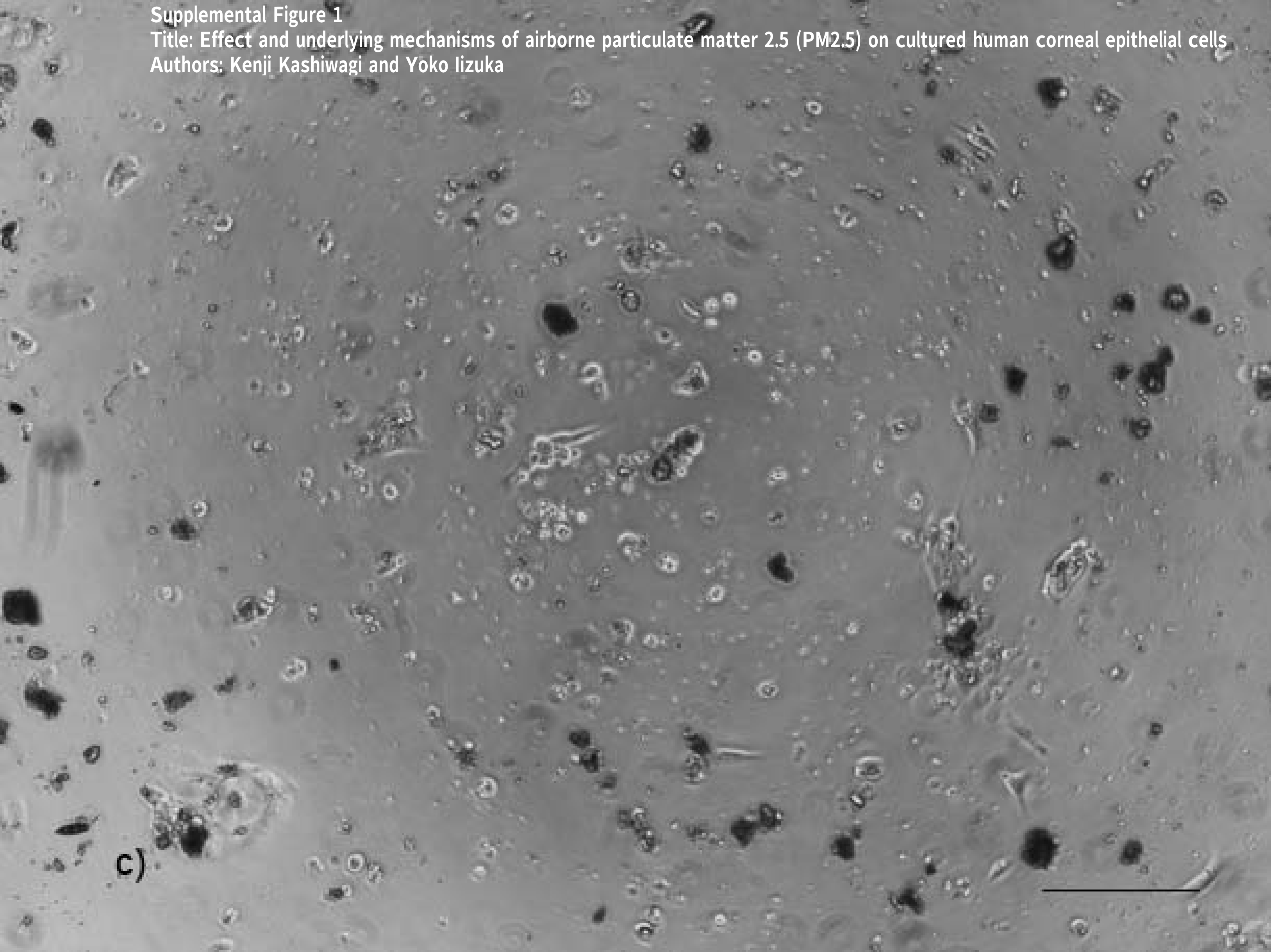

Supplemental Figure 1

Title: Effect and underlying mechanisms of airborne particulate matter 2.5 (PM2.5) on cultured human corneal epithelial cells

Authors: Kenji Kashiwagi and Yoko Iizuka

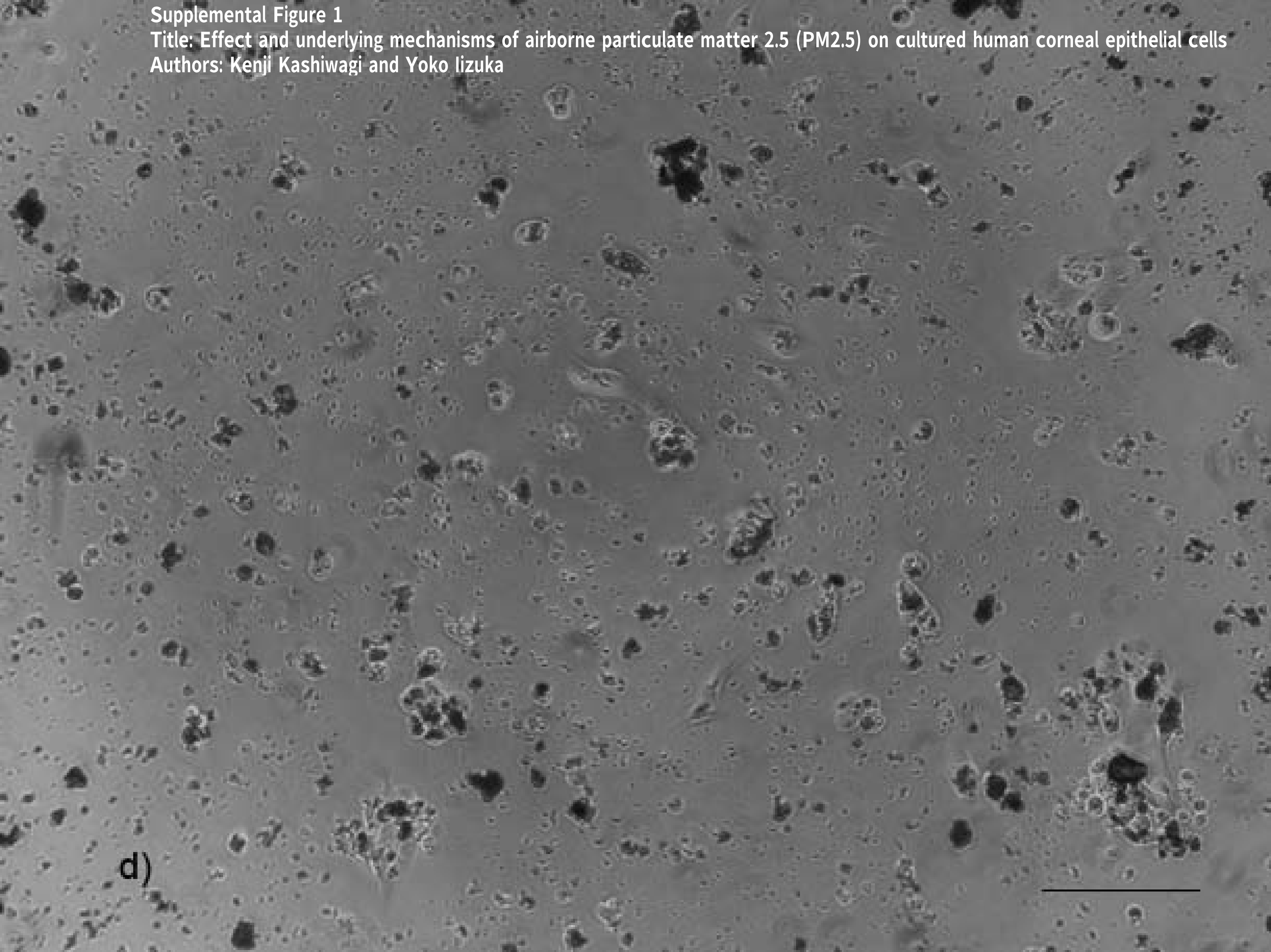

Supplemental Figure 1
Title: Effect and underlying mechanisms of airborne particulate matter 2.5 (PM2.5) on cultured human corneal epithelial cells
Authors: Kenji Kashiwagi and Yoko Iizuka

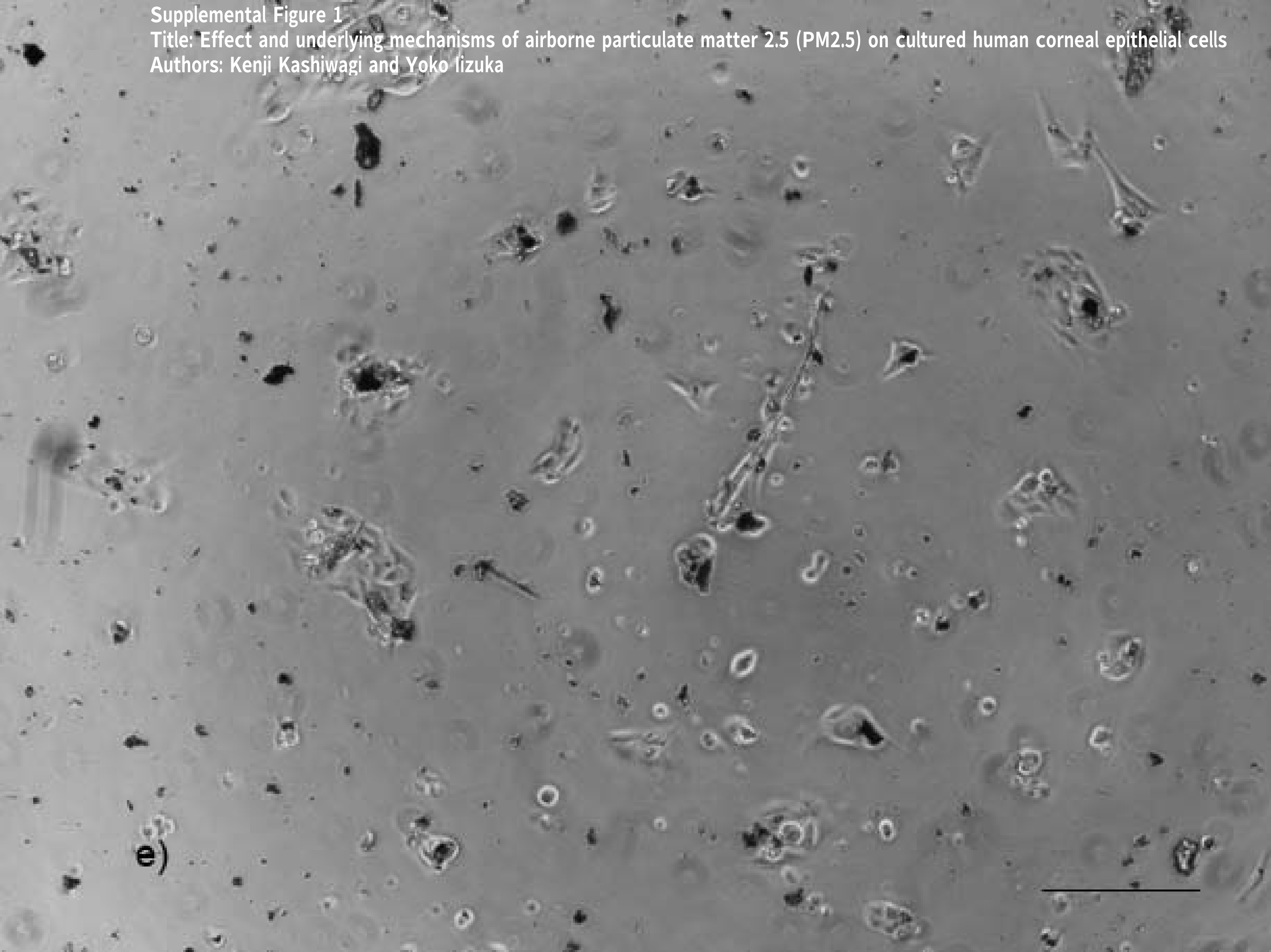

Supplemental Figure 1
Title: Effect and underlying mechanisms of airborne particulate matter 2.5 (PM2.5) on cultured human corneal epithelial cells
Authors: Kenji Kashiwagi and Yoko Iizuka

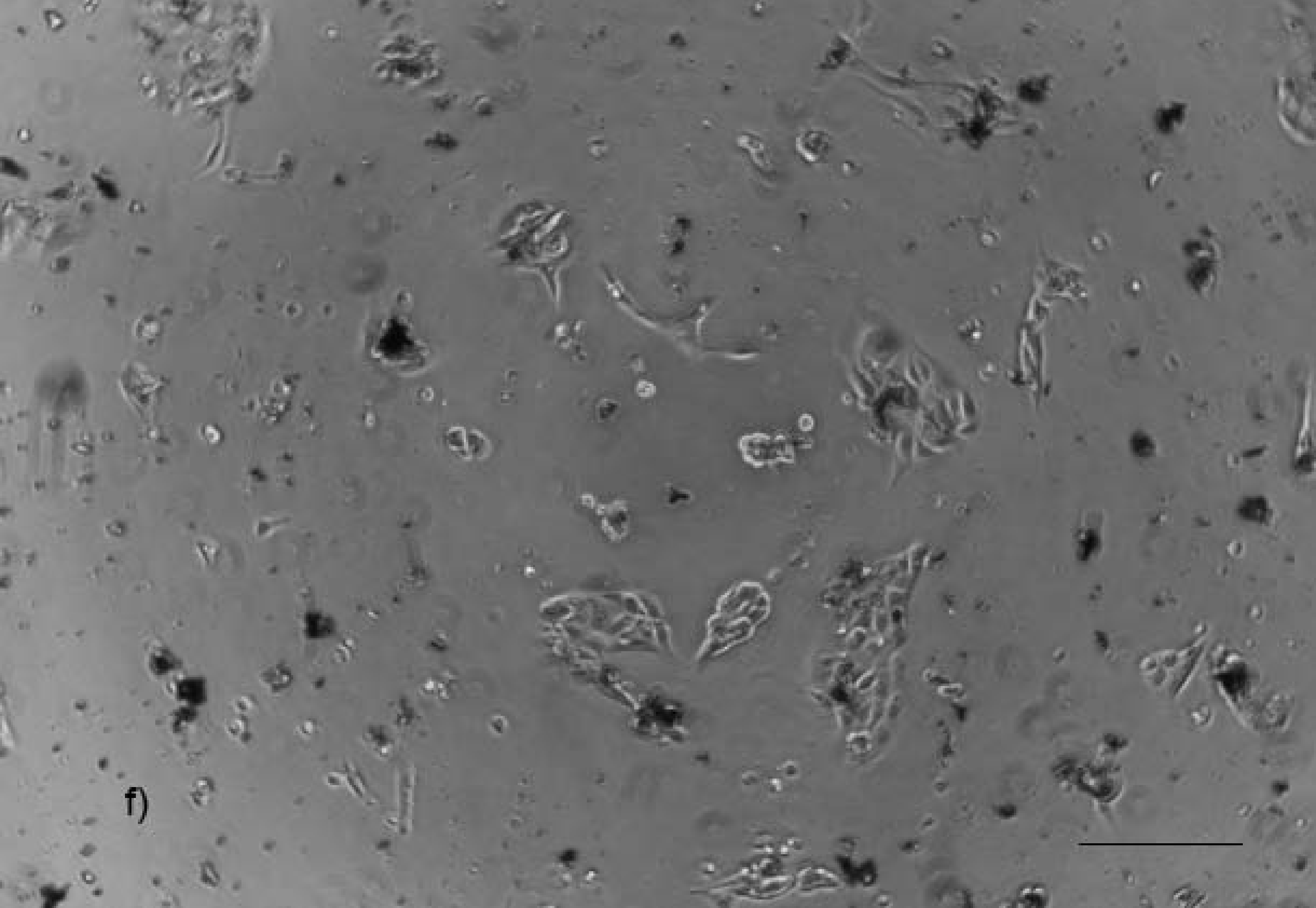

Supplemental Figure 1
Title: Effect and underlying mechanisms of airborne particulate matter 2.5 (PM2.5) on cultured human corneal epithelial cells
Authors: Kenji Kashiwagi and Yoko Iizuka

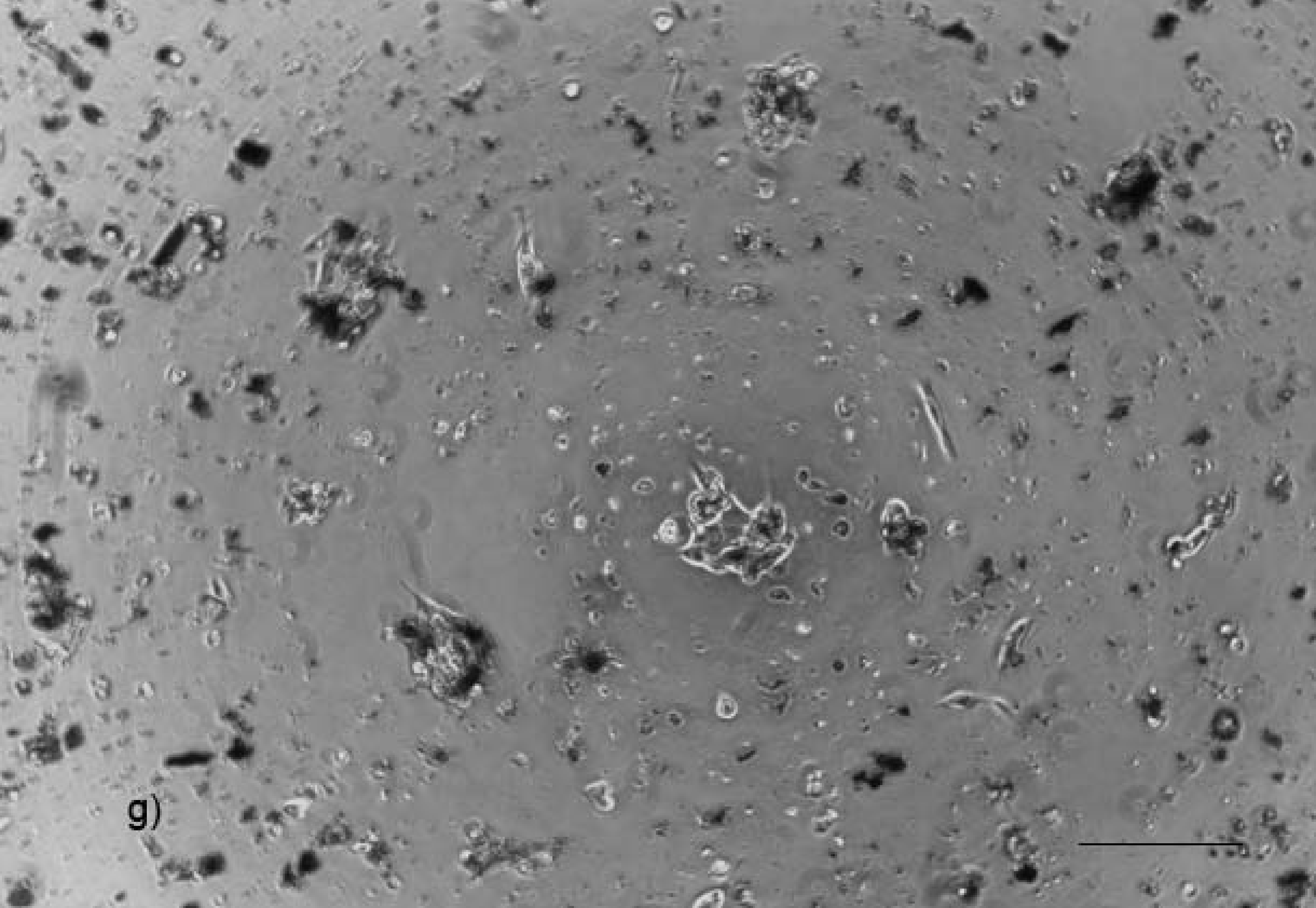

Supplemental Figure 1
Title: Effect and underlying mechanisms of airborne particulate matter 2.5 (PM2.5) on cultured human corneal epithelial cells
Authors: Kenji Kashiwagi and Yoko Iizuka

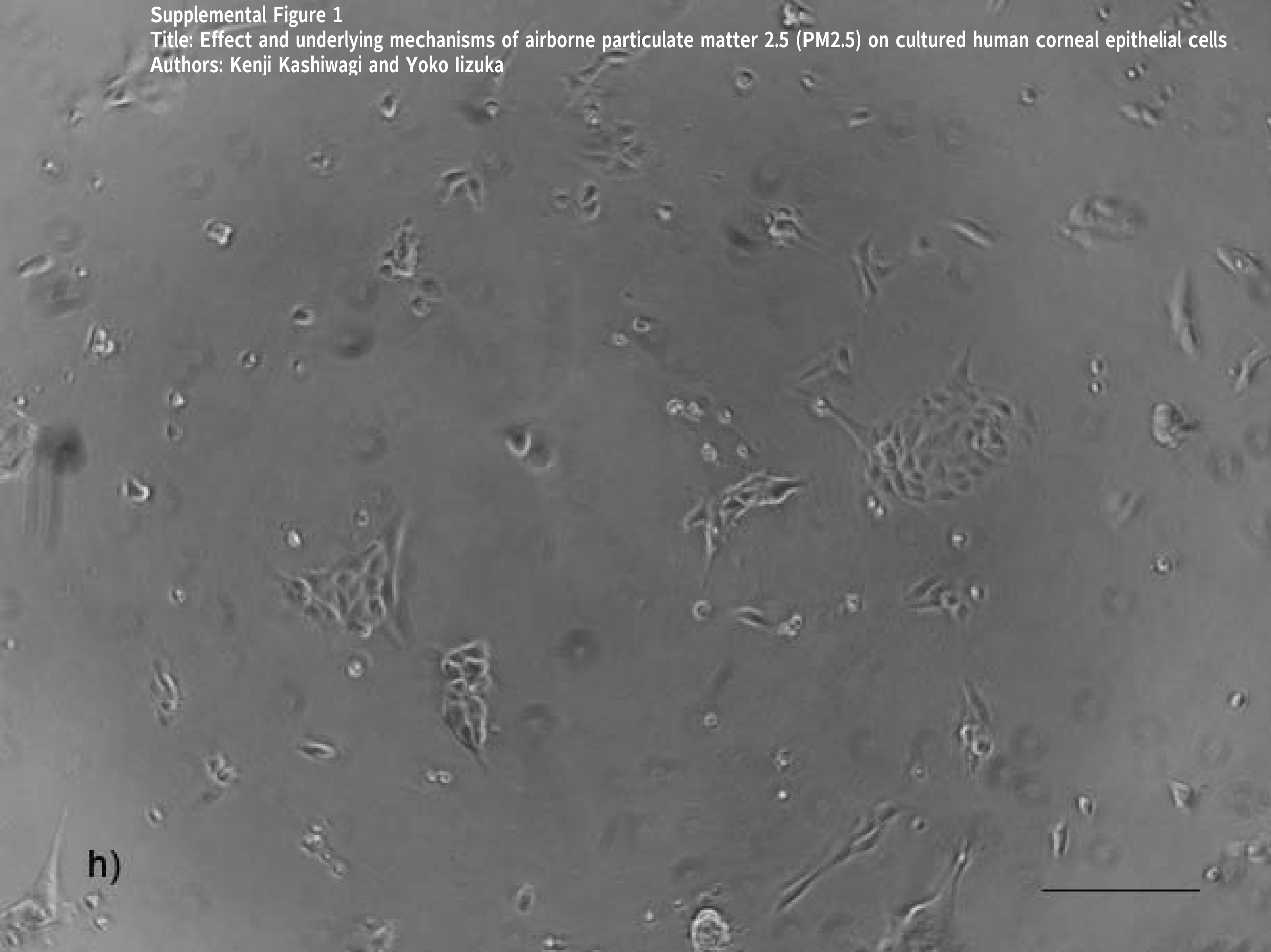

Supplemental Figure 1
Title: Effect and underlying mechanisms of airborne particulate matter 2.5 (PM2.5) on cultured human corneal epithelial cells
Authors: Kenji Kashiwagi and Yoko Iizuka

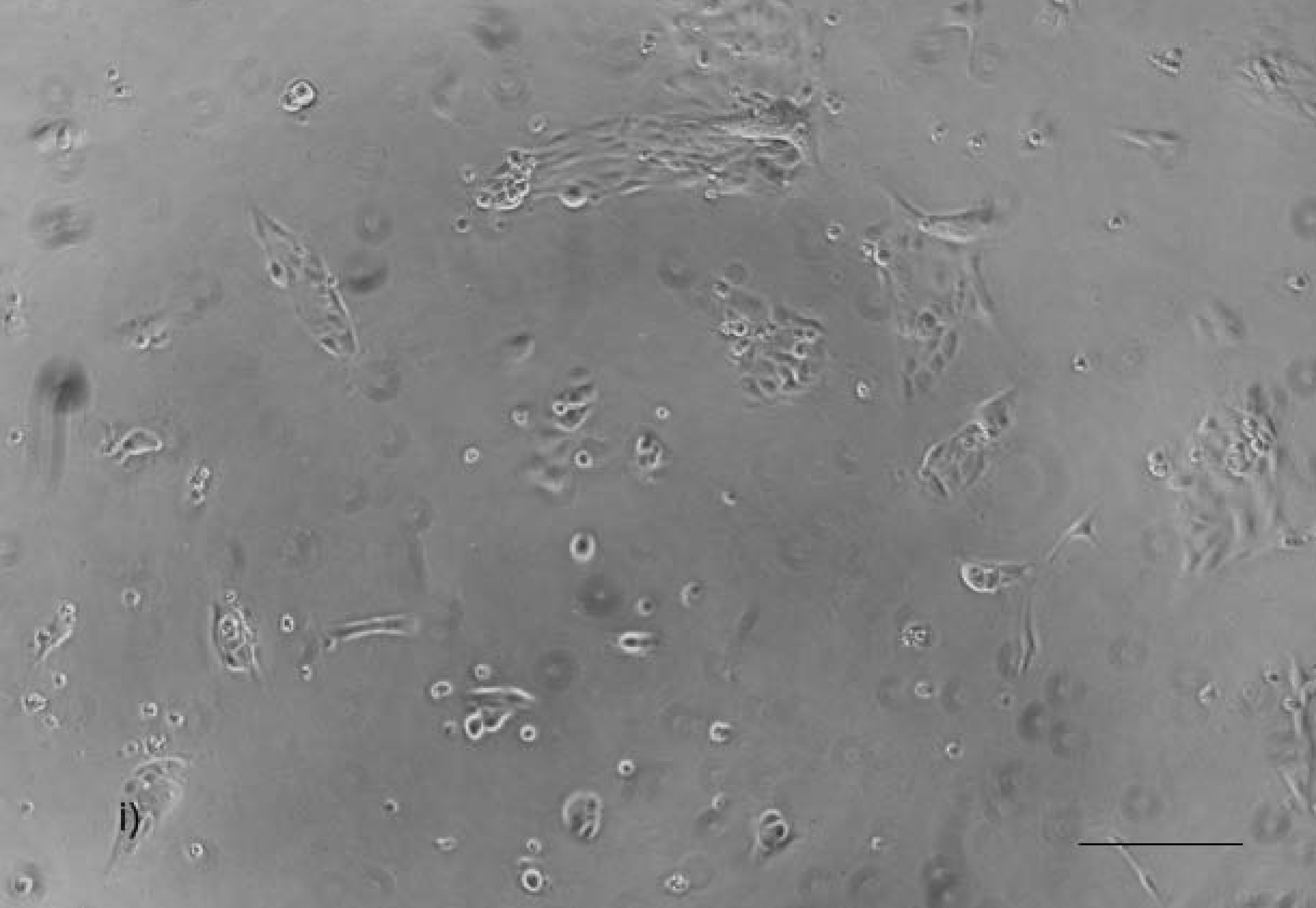

Supplemental Table 1
Title: Effect and underlying mechanisms of airborne particulate matter 2.5 (PM2.5) on cultured human corneal epithelial cells
Authors: Kenji Kashiwagi and Yoko Iizuka

| **Material** | **unit** | **#30** | **# 28** |
|---|---|---|---|
| Na | % | 0.939±0.071 | 0.796±0.065 |
| Mg | % | 1.51±0.13 | 1.4±0.06 |
| Al | % | 7.58±0.42 | 5.04±0.1 |
| K | % | 2.13±0.11 | 1.37±0.06 |
| Ca | % | 4.25±0.35 | 6.69±0.24 |
| Ti | % | 0.426±0.04 | 0.292±0.033 |
| Fe | % | 3.84±0.35 | 2.92±0.17 |
| Mn | mg/kg | 768±83 | 686±42 |
| Zn | mg/kg | 93.1±8.5 | 0.114±0.01 |
| Sr | mg/kg | 250±20 | 469±16 |
| Ba | mg/kg | 535±31 | 874±65 |
| V | mg/kg | ND | 73.2±7 |
| Ni | mg/kg | 29.1* | 63.8±3.4 |
| Cu | mg/kg | ND | 104±12 |
| As | mg/kg | ND | 90.2±10.7 |
| Cd | mg/kg | ND | 5.6±0.43 |
| Pb | mg/kg | 22.4* | 403±32 |
| U | mg/kg | 2.62* | 4.33±0.26 |
| Si | % | 24.1* | 14.9* |
| P | mg/kg | 955* | 0.145* |
| Sc | mg/kg | 13.1* | 10.7* |
| Cr | mg/kg | 57.4* | 65.6* |
| Co | mg/kg | 13.7* | 22* |
| Cl | % | ND | 0.807* |
| La | mg/kg | 40.4* | 32.7* |
| Be | mg/kg | ND | 5.09* |
| Th | mg/kg | 13* | 11.1* |
| Se | mg/kg | ND | 14.4* |
| Rb | mg/kg | ND | 64.1* |
| Y | mg/kg | ND | 21.9* |
| Mo | mg/kg | ND | 28.4* |
| Sn | mg/kg | ND | 21.5* |
| Sb | mg/kg | ND | 20.1* |
| S | % | ND | 3.91* |

Supplemental Figure 2
Title: Effect and underlying mechanisms of airborne particulate matter 2.5 (PM2.5) on cultured human corneal epithelial cells
Authors: Kenji Kashiwagi and Yoko Iizuka

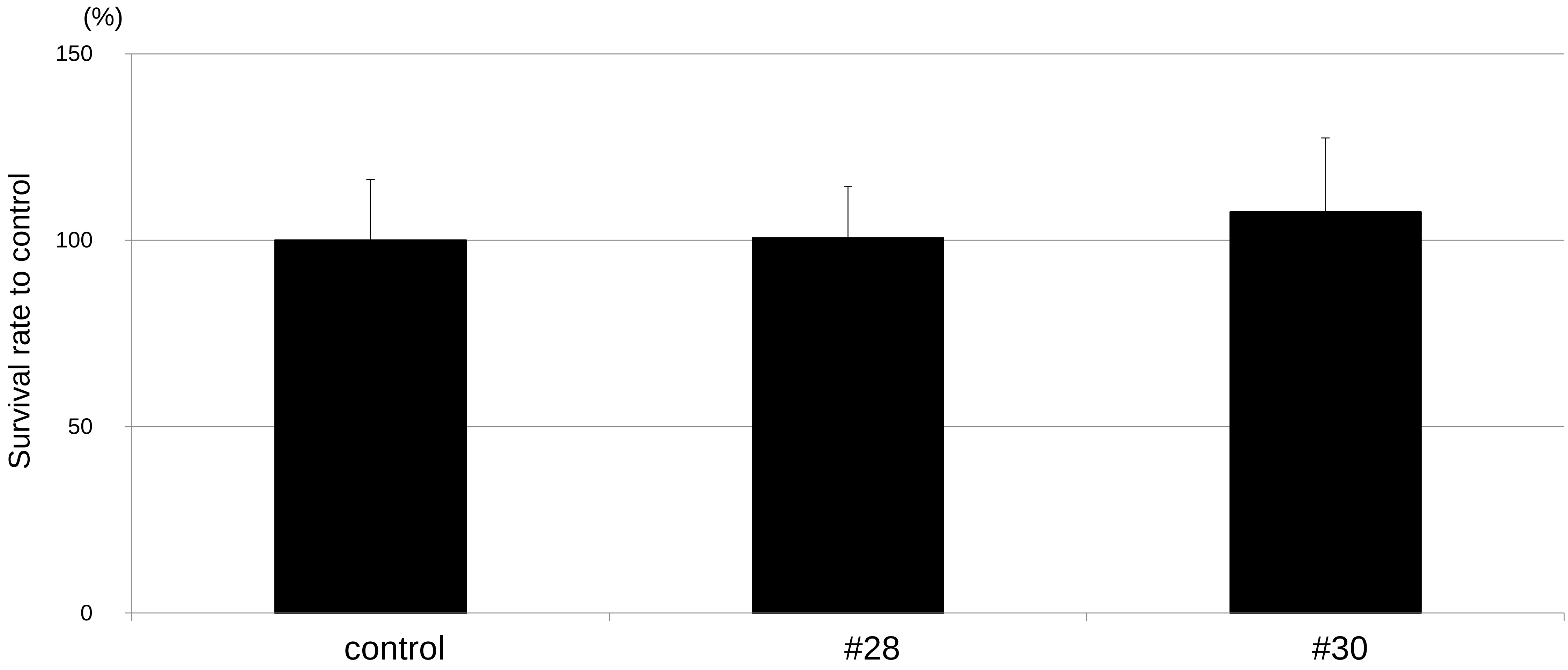

Supplemental Table 2

Title: Effect and underlying mechanisms of airborne particulate matter 2.5 (PM2.5) on cultured human corneal epithelial cells

Authors: Kenji Kashiwagi and Yoko Iizuka

| Cytokine (pg/ml) | Control | #28 | #30 |
|---|---|---|---|
| Epidermal growth factor | < OOR | < OOR | < OOR |
| Fibroblast growth factor-2 | < OOR | < OOR | < OOR |
| Eotaxin | < OOR | < OOR | < OOR |
| Transforming growth factor-α | 1.76* | 1.65* | 1.37* |
| granulocyte-colony stimulating factor | < OOR | < OOR | < OOR |
| Feline McDonough Sarcoma-related tyrosine kinase 3 | < OOR | < OOR | < OOR |
| Granulocyte macrophage colony-stimulating factor | 52.57 | 49.31 | 60.71 |
| Fractalkine | < OOR | < OOR | < OOR |
| Interferon (IFN) -α2 | < OOR | < OOR | < OOR |
| IFN gammna | < OOR | < OOR | < OOR |
| Growth-regulated oncogene | < OOR | < OOR | < OOR |
| Interleukin (IL)-10 | < OOR | < OOR | < OOR |
| Monocyte chemotactic protein (MCP)-3 | < OOR | < OOR | < OOR |
| IL-12p40 | < OOR | < OOR | < OOR |
| Macrophage-derived chemokine | < OOR | < OOR | < OOR |
| IL-12p70 | < OOR | < OOR | < OOR |
| IL-13 | < OOR | < OOR | < OOR |
| IL-15 | < OOR | < OOR | < OOR |
| Soluble cluster of differentiation (CD)40L | < OOR | < OOR | < OOR |
| IL-17A | 0.39* | 0.39* | 0.6* |
| IL-1RA | < OOR | < OOR | < OOR |
| IL-1alpha | < OOR | < OOR | 1.38* |
| IL-9 | < OOR | < OOR | < OOR |
| IL-1beta | < OOR | < OOR | < OOR |
| IL-2 | 0.88 | 0.88 | 0.57* |
| IL-3 | 0.43* | 0.43* | 0.38* |
| IL-4 | < OOR | < OOR | < OOR |
| IL-5 | < OOR | < OOR | < OOR |
| IL-6 | 67.49 | 49.83 | 23.33 |
| IL-7 | < OOR | < OOR | < OOR |
| IL-8 | 23.08 | < OOR | < OOR |
| Interferon-gamma inducible protein-10 | 9.38* | 6.77* | 8.12* |
| MCP-1 | 24.99 | < OOR | < OOR |
| Macrophage inflammatory protein (MIP)-1α | 0.94* | 0.6* | < OOR |
| MIP-1beta | < OOR | < OOR | < OOR |
| Tumor necrosis factor (TNF)-α | 0.78 | 0.78 | < OOR |
| TNF-β | < OOR | < OOR | < OOR |
| Vascular endothelial growth factor | < OOR | < OOR | < OOR |
