# Supplementary material for: Effect and underlying mechanisms of airborne particulate matter 2.5 (PM2.5) on cultured human corneal epithelial cells
Source: Sci Rep. 2020 Nov 11;10:19516. doi: 10.1038/s41598-020-76651-9 (PMC7659009; doi:10.1038/s41598-020-76651-9)
Supplement: Supplementary file 2 — Supplementary Information 2. [file 41598_2020_76651_MOESM2_ESM.docx]

**Supplemental Figure 1 PM2.5 induces morphological changes in HCECs**

Images of HCECs after 24 hours of PM2.5 exposure. The number of HCECs showing white spindles or round shapes decreased, and cell body contraction occurred in a concentration-dependent manner. The black particles are PM2.5. The arrows indicate representative particles. In contrast, increases in cell number and aggregation were observed in HCECs exposed to LPS. a) Control, b) 50 µg/ml #30, c) 100 µg/ml #30, d) 200 µg/ml #30, e) 50 µg/ml #28, f) 100 µg/ml #28, g) 200 µg/ml #28, h) 100 µg/ml LPS, and i) 200 µg/ml LPS. LPS: lipopolysaccharide. Scale bar=50 µm.

**Supplemental Table 1 Comparison of the components of the two types of PM2.5 used in the current study**

ND: not detectable, *unreliable.

**Supplemental Figure 2 Effect of the coculture system on HCEC survival**

**Supplemental Table 2 Concentrations of the cytokines released into the culture medium**

<OOR: smallest detectable concentration, * unreliable measurement.
